# Dimethylsulfoxide Inhibits Oligodendrocyte Fate Choice of Adult Neural Stem and Progenitor Cells

**DOI:** 10.3389/fnins.2019.01242

**Published:** 2019-11-26

**Authors:** Anna O’Sullivan, Simona Lange, Peter Rotheneichner, Lara Bieler, Ludwig Aigner, Francisco J. Rivera, Sebastien Couillard-Despres

**Affiliations:** ^1^Institute of Experimental Neuroregeneration, Paracelsus Medical University, Salzburg, Austria; ^2^Spinal Cord Injury and Tissue Regeneration Center Salzburg (SCI-TReCS), Salzburg, Austria; ^3^Department of Otorhinolaryngology, Paracelsus Medical University, Salzburg, Austria; ^4^Institute of Molecular Regenerative Medicine, Paracelsus Medical University, Salzburg, Austria; ^5^Austrian Cluster for Tissue Regeneration, Vienna, Austria; ^6^Laboratory of Stem Cells and Neuroregeneration, Institute of Anatomy, Histology and Pathology, Faculty of Medicine, Universidad Austral de Chile, Valdivia, Chile; ^7^Center for Interdisciplinary Studies on the Nervous System (CISNe), Universidad Austral de Chile, Valdivia, Chile

**Keywords:** oligodendrogenesis, neural stem cells, myelination, DMSO, Id2, Olig2, astrocyte, oligodendrocytes

## Abstract

Several clinical trials address demyelinating diseases via transplantation of mesenchymal stromal cells (MSCs). Published reports detail that administration of MSCs in patients may provide a beneficial immunomodulation, and that factors secreted by MSCs are potent inducers of oligodendrogenesis. Dimethylsulfoxide (DMSO) is widely used in life science and medicine as solvent, vehicle or cryoprotectant for cells used in transplantation. Importantly, most transplantation protocols do not include the removal of DMSO before injecting the cell suspension into patients. This indifferent application of DMSO is coming under increasing scrutiny following reports investigating its potential toxic side-effects. While the impact of DMSO on the central nervous system (CNS) has been partially studied, its effect on oligodendrocytes and oligodendrogenesis has not been addressed yet. Consequently, we evaluated the influence of DMSO on oligodendrogenesis, and on the pro-oligodendrogenic effect of MSCs’ secreted factors, using adult rat neural stem and progenitor cells (NSPCs). Here, we demonstrate that a concentration of 1% DMSO robustly suppressed oligodendrogenesis and drove the fate of differentiating NSPCs toward astrogenesis. Furthermore, the pro-oligodendrogenic effect of MSC-conditioned medium (MSCCM) was also nearly completely abolished by the presence of 1% DMSO. In this condition, inhibition of the Erk1/2 signal transduction pathway and high levels of Id2 expression, a specific inhibitor of oligodendrogenic differentiation, were detected. Furthermore, inflammatory demyelinating diseases may even potentiate the impact of DMSO on oligodendrogenesis. Our results demonstrate the imperative of considering the strong anti-oligodendrogenic activity of DMSO when designing future clinical trial protocols.

## Introduction

In young adults, the most common neurological disease leading to permanent disability is multiple sclerosis (MS). The latter is a neuroinflammatory disorder of the central nervous system (CNS) characterized by the progressive destruction of myelin sheaths. Although MS patients can recover from their first demyelinating episodes through a process known as remyelination, the regenerative capacity of the CNS declines through the course of the disease. This leads to physical and cognitive deficits over time due to insufficient axon insulation and neuronal loss. Despite new drug developments, the repair of CNS damage still constitutes a challenging problem (Wingerchuk and Carter, 2014). As our understanding of the mechanisms underlying the process of remyelination grows, there is increasing interest in cell-based therapies to address the issue.

We previously reported that factors secreted by mesenchymal stromal cells (MSCs) induce oligodendrogenic fate decision in neural stem and progenitor cells (NSPCs) and oligodendrocyte precursor cells (OPCs) (Rivera et al., 2006, 2008, 2019; Jadasz et al., 2013), both of which represent natural sources for new oligodendrocytes in the CNS (Menn et al., 2006; Franklin and Ffrench-Constant, 2008; Etxeberria et al., 2010; Jablonska et al., 2010; Kazanis et al., 2017). Furthermore, MSCs are potent immunomodulators (Uccelli and Mancardi, 2010), are easily accessible from the bone marrow, and their administration has been proven safe (Dahbour et al., 2017).

These properties encouraged the development of multiple clinical trials involving the application of MSCs to patients suffering from a demyelinating disease (summary of published studies of MSC transplantation in MS in Scolding et al., 2017). However, diverging results between studies raise questions about the efficacy of this MSC-based strategy and warrant standardized protocols, which should include the origin of cell product, the route of delivery, the dose, and the trial design (Scolding et al., 2017). Consideration should also be given to the presence or removal of the cryoprotectant dimethylsulfoxide (DMSO) from the cell preparation administered. While it has been demonstrated that a standard dose of 10% DMSO in the cell suspension administered is generally well tolerated, adverse reactions to cell applications could significantly be reduced when DMSO was removed or its concentration reduced prior to injection (Windrum et al., 2005). Moreover, case reports describing neurological adverse events like epileptic seizures, stroke, encephalopathy or leukoencephalopathy, following administration of DMSO suggest that it might be harmful for the CNS (Dhodapkar et al., 1994; Higman et al., 2000; Hoyt et al., 2000; Bauwens et al., 2005). Previous studies explored the influence of DMSO on neurons and astrocytes and reported concerning results about cell toxicity even at low DMSO concentrations (Hanslick et al., 2009; Tamagnini et al., 2014; Yuan et al., 2014; Zhang et al., 2017). However, DMSO’s effect on other CNS resident precursor cells has not been addressed yet.

Our study demonstrates that a concentration of 1% DMSO almost completely obliterates the generation of oligodendrocytes from adult rat NSPCs in favor of astrocytic differentiation. These results substantiate the assumption that DMSO could have a negative impact on oligodendrogenesis, and we therefore call for further investigation of its potential hazard when applied in the context of demyelinating disorders.

## Materials and Methods

### Animal Works

Experiments were performed in accordance to the guidelines of the “Directive 2010/63/EU of the European Parliament and of the Council of 22 September 2010 on the protection of animals used for scientific purposes.” According to the European Directive 2010/63/EU Article 3 and the Austrian legislation for experiments on living animals Tierversuchsgesetz 2012 §2c, no additional approval was required for the killing of animals with the aim of collecting tissues.

### MSC Cultures and MSCCM

Mesenchymal stromal cell cultures and MSC-conditioned medium (MSCCM) were generated as described by Rivera et al. (2006). In brief, bone marrow plugs from tibias and femurs of 6–8 weeks old female Fischer-344 rats were harvested and mechanically dissociated in α-MEM. After centrifugation and resuspension, dissociated bone marrow cells were seeded at 1 × 10^6^ cells/cm^2^ in α-MEM containing 10% FBS, 100 U/mL penicillin and 100 μg/mL streptomycin (thereafter aMEM) and grown in a humidified incubator at 37°C with 5% CO_2_. Medium was changed every third day until a confluent cell layer was reached. For MSCCM production, MSCs were seeded at a density of 12,000 cells/cm^2^ in aMEM. After 3 days of incubation, the supernatant (conditioned medium) was collected and filtered using a 0.22 μm pore filter (Rivera et al., 2006).

### NSPC Cultures

Neural stem and progenitor cells cultures from the adult hippocampus (HC) and subventricular zone (SVZ) were established as described by Wachs et al. (2003). Briefly, 6–8 weeks old female Fischer-344 rats were decapitated and their HC and SVZ dissected. The tissue was transferred to 4°C cold PBS containing 4.5 mg/L D-glucose, then mechanically minced and enzymatically digested. Dissociated cells were collected and resuspended in Neurobasal A medium (NBA, Gibco) supplemented with B27 (Gibco), 2 mM L-glutamine, 100 U/mL penicillin, 100 μg/mL streptomycin, as well as 2 μg/mL heparin, 20 ng/mL human recombinant EGF and 20 ng/mL human recombinant FGF (thereafter referred to as NBA+all). Neurosphere cultures were kept in a humidified incubator with 5% CO_2_ at 37°C and half of the medium was changed every third day.

For passaging, the medium containing floating neurospheres was centrifuged at 120 × *g* for 5 min. The supernatant was discarded and the pellet dissociated with Accutase (PAN Biotech). The single cells were then reseeded into NBA+all at a density of 1 × 10^5^ cells/mL. Passaging took place every 7–9 days.

For freezing, the cell suspension was centrifuged 3 days after passaging and the supernatant was discarded. The cells were then transferred into cryomedium (10% DMSO, 20% FBS, 70% NBA supplemented with B27, 2 mM L-glutamine, 100 U/mL penicillin, 100 μg/mL streptomycin) and frozen at −150°C until further use. After thawing, cells were immediately washed with NBA+all to remove remnants of the cryomedium, and cultured in NBA+all.

All NSPCs used in the experiments were frozen at passage 1 or 2, and employed for the various experiments in passage number 2–6. No difference in fate choice was observed during differentiation between fresh cells, and cultures derived from frozen stocks (data not shown). Data provided in the main figures were generated with hippocampal NSCs. Differentiation of NSCs obtained from the SVZ was not significantly different (Supplementary Figure S1).

### Treatment of NSPCs

Neurospheres were dissociated using Accutase (PAN Biotech) and seeded onto 100 μg/mL poly-L-ornithine and 5 μg/mL laminin coated coverslips at a density of 8000–10,000 cells/cm^2^ in aMEM. After 16 h, medium was replaced with aMEM or MSCCM containing the final concentration of DMSO. After 3 or 6 days of differentiation, the NSPCs were fixed with 4% paraformaldehyde and processed for immunofluorescence stainings.

For RNA-isolation, NSPCs were grown as mentioned above on poly-L-ornithine and laminin coated petri dishes in aMEM and MSCCM containing either 0 or 1% of DMSO. After 3 days of differentiation, NSPCs were lysed with Tri Reagent (Sigma-Aldrich) and total RNA was isolated using the RNeasy plus mini RNA isolation kit (Qiagen) (see below).

For protein isolation, NSPCs were seeded onto poly-L-ornithine and laminin coated petri dishes for 16 h. After that, the NSPCs were stimulated with aMEM or MSCCM with 0 or 1% DMSO. After 10 min of treatment, protein isolation was performed using a RIPA-buffer.

### Immunofluorescence Analysis and Quantification

Immunofluorescence analysis and quantification were performed as described by Oberbauer et al. (2013). Following primary antibodies and concentrations were used: guinea pig anti-Glial Acidic Fibrillary Protein (GFAP) 1:500 (Progen); mouse anti-2′,3′-cyclic-nucleotide-3′-phosphodieesterase (CNP) 1:200 (Millipore); rabbit anti-Caspase3 (Casp3) 1:200 (Abcam); goat anti-oligodendrocyte transcription factor 2 (Olig2) 1:20 (R&D Systems); mouse anti-neural/glial antigen 2 (NG2) 1:500 (Sigma-Aldrich). Secondary antibodies: donkey anti-guinea pig Alexa Fluor 647 1:500 (Dianova); donkey anti-rabbit Alexa Fluor 488 1:1000 (Thermo Fisher Scientific); donkey anti-mouse Alexa Fluor 568 1:1000 (Thermo Fisher Scientific). Nuclear counterstaining was performed with 0.25 μg/mL 4′,6′-diamidino-2-phenylindole dihydrochloride hydrate (DAPI; Sigma). Epifluorescence was observed using an Olympus IX81 (Olympus) equipped with a Hamamatsu digital camera (Hamamatsu Photonics) and CellSense Software (Olympus) for documentation. Ten observation fields were chosen randomly, containing between 300 and 1300 cells, and photographed for analysis of cell fate decision.

### Protein Extraction and Western Blots

Neural stem and progenitor cells were treated as mentioned above and after washing with PBS, cells were lysed with RIPA buffer supplemented with protease inhibitors (cOmplete, Roche) and phosphatase inhibitors (PhosSTOP, Roche). The suspensions were homogenized through a 27-gauge needle and samples placed on ice for 30 min. Protein concentrations of lysates were determined with a Pierce BCA Protein Assay Kit (Thermo Fisher Scientific). Equal amounts (10 μg) of protein samples were loaded on Mini-PROTEAN TGX Stain-free precast gel (Bio-Rad) and run with 120 V. Precision Plus Protein Dual Color Standard (Bio-Rad) as well as SeeBlue Plus2 Pre-stained Protein Standard (Thermo Fisher Scientific) were used as protein standard markers. Blotting was performed using a Trans-Blot Turbo Transfer System (Bio-Rad) with Trans-Blot Turbo Mini Transfer Packs (Bio-Rad).

Membranes were blocked using 5% BSA in Tris–buffered saline with 0.1% Triton X-100 (TBST) for 1 h at room temperature prior to incubation with the primary antibodies diluted in the same buffer overnight at 4°C. Following primary antibodies were used: rabbit anti-Erk1/2 1:1000 (p44/42 MAPK (Erk1/2) mAb #4695, Cell Signaling Technologies); rabbit anti-pErk1/2 1:1000 (Phospho-p44/42 MAPK (Erk1/2) (Thr202/Tyr204), mAb #4370, Cell Signaling Technologies); mouse anti-beta actin 1:2000 (Sigma). The membranes were then washed with TBST and incubated with secondary antibodies diluted in blocking solution for 2 h at room temperature: donkey anti-rabbit Alexa Fluor 568 1:1000 (Thermo Fisher Scientific); donkey anti-mouse Alexa Fluor 488 1:1000 (Thermo Fisher Scientific). Immunocomplexes were visualized using a Chemidoc (Bio-Rad) and the Bio-Rad ImageLab software was used for analysis and quantification.

### Quantitative Gene Expression Analyses

Neural stem and progenitor cells were differentiated for 3 days as mentioned above. RNA-extractions were performed using TRI Reagent (Sigma) followed by the use of an RNeasy plus mini RNA isolation kit (Qiagen). The cDNA synthesis was performed with iScript Reverse Transcription Supermix for RT-qPCR (Bio-Rad). Quantitative gene expression analyses were performed using TaqMan RT-PCR technology. Technical duplicates containing 10 ng of cDNA were amplified with the GoTAQ Probe qPCR Master Mix (Promega) using a two-step cycling protocol (95°C for 15 s, 60°C for 60 s; 40 cycles, Bio-Rad CFX 96 Cycler).

The following gene expression assays were employed: Id2 (Rn01495280_m1, Applied Biosystems), Olig2 (Rn0056603_m1, Applied Biosystems), DCX (Rn00584505_m1, Applied Biosystems) as well as the following housekeepers: Hprt1 (Rn01527840_m1, Applied Biosystems), Sdha (Rn00590475_m1, Applied Biosystems), Heatr3 (Rn.PT.56a.5120057, Integrated DNA Technologies, United States); TBP (Rn.PT.39a.22214837, Integrated DNA Technologies, United States) Ywhaz (Rn.PT.56a.8368619, Integrated DNA Technologies, United States). Only validated housekeepers were included in the analysis.

Quantification analyses were performed with qBase Plus (Biogazelle, Belgium) using geNorm algorithms for multi- reference gene normalization followed by normalization to average.

### Statistical Analysis

Data are presented as means ± SD. Statistical analysis was performed using RStudio (RStudio Inc., Boston, MA, United States). Data sets were analyzed according to the general factorial design included in the GFD-package in R (Friedrich et al., 2017) using the ANOVA-type approximation for the calculation of *p*-values. *P*-values of <0.05 were considered significant. Significance: ns, *p* ≥ 0.05; ^∗^*p* < 0.05; ^∗∗^*p* < 0.01; ^∗∗∗^*p* < 0.001.

## Results

### DMSO Inhibits Oligodendrogenic Fate Decision of NSPCs

**FIGURE 1:**
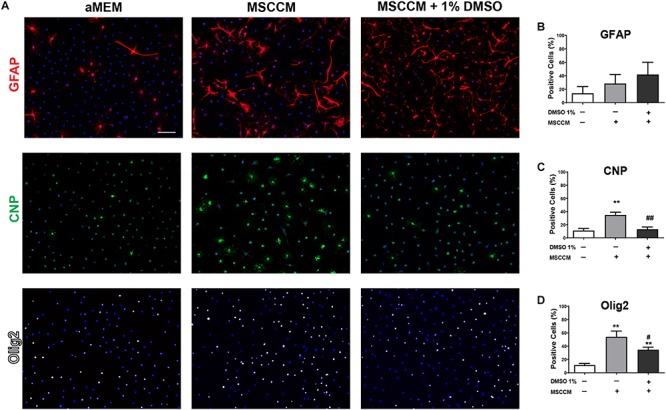
Impact of MSCCM and DMSO on early differentiation of NSPCs (3 days). **(A)** Representative immunodetection of markers for astrocytes (GFAP, red), oligodendrocytes (CNP, green) and cells of the oligodendrocytic lineage (Olig2, white) in NSPCs under the various differentiation conditions for 3 days. Nuclei were counterstained with DAPI (blue). Scale bar = 100 μm. Quantification of the number of cells expressing **(B)** GFAP, **(C)** CNP, and **(D)** Olig2 after 3 days of differentiation. Data represented as mean ± SD. Asterisks mark significant differences in relation to aMEM, pound signs mark significant differences in relation to MSCCM, ^∗∗^*p* < 0.01, #*p* < 0.05, ##*p* < 0.01.

Novel strategies for remyelination strive to take advantage of the pro-oligodendrogenic effect of MSCs, while the mechanisms behind this activity are still being resolved. As DMSO is frequently used as a vehicle, solvent or cryoprotectant for cells, we analyzed its influence on the pro-oligodendrogenic properties of MSCs. NSPCs were incubated during differentiation in aMEM, MSCCM or MSCCM containing 1% of DMSO. After 3 days in these differentiation conditions, the expression of GFAP (astrocytes), CNP (oligodendrocytes) and Olig2 (entire oligodendroglial lineage) were examined by immunohistology (Figure 1). While the expression of GFAP was not different in aMEM and MSCCM (aMEM: 14.0 ± 10.0% and MSCCM: 28.4 ± 13.5%, *p* = 0.216, *n* = 3), differences in CNP expression were already evident after 3 days of differentiation (aMEM: 11.2 ± 3.1% and MSCCM: 34.9 ± 4.2%, *p* = 0.002, *n* = 3). Addition of 1% DMSO to MSCCM did not show significant changes in the number of GFAP-expressing cells (MSCCM + 1% DMSO: 42.0 ± 18.1%, compared to MSCCM: *p* = 0.361 or aMEM *p* = 0.097, *n* = 3). In contrast, following application of 1% DMSO, the percentage of CNP-expressing oligodendrocytes was substantially reduced compared to MSCCM (MSCCM + 1% DMSO: 13.2 ± 3.1%, *p* = 0.003, *n* = 3). Expression of Olig2, a lineage marker for oligodendrocytes, robustly increased in the presence of MSCCM (MSCCM: 54.1 ± 8.6% compared aMEM: 11.7 ± 2.5%, *p* = 0.009, *n* = 3). The addition of 1% DMSO to MSCCM significantly reduced the percentage of cells expressing Olig2, however, their abundance was still significantly higher than in aMEM (MSCCM + 1% DMSO: 34.5 ± 4.0%, compared to MSCCM: *p* = 0.041 compared aMEM: *p* = 0.002, *n* = 3).

We evaluated the expression of the neurogenic marker DCX after 3 days of differentiation in aMEM and MSCCM, with or without 1% DMSO. As previously described, neuronal differentiation of adult rat NSPCs in aMEM and MSCCM takes place at very low levels (Rivera et al., 2006; Couillard-Despres et al., 2008; Ladewig et al., 2008; Steffenhagen et al., 2012) and expression of the neuronal markers Map2ab or DCX remain in the low single-digit percentages. We therefore addressed the impact of DMSO on neuronal differentiation based on the expression of DCX mRNA levels. Addition of 1% DMSO to aMEM significantly decreased the relative expression of DCX mRNA (aMEM: 2.92 ± 0.82 to aMEM + 1% DMSO 1.30 ± 0.44, *p* = 0.037, *n* = 3). Furthermore, also MSCCM had a detrimental effect on DCX expression compared to aMEM (MSCCM: 0.48 ± 0.10, *p* = 0.034, *n* = 3). The addition of DMSO to MSCCM did not further decrease DCX mRNA relative expression (MSCCM + 1% DMSO: 0.59 ± 0.11, *p* = 0.285, *n* = 3) (see Supplementary Figure S2). Hence, in the absence of a neuronal induction stimuli, the influence of DMSO on neuronal differentiation remained marginal.

**FIGURE 2:**
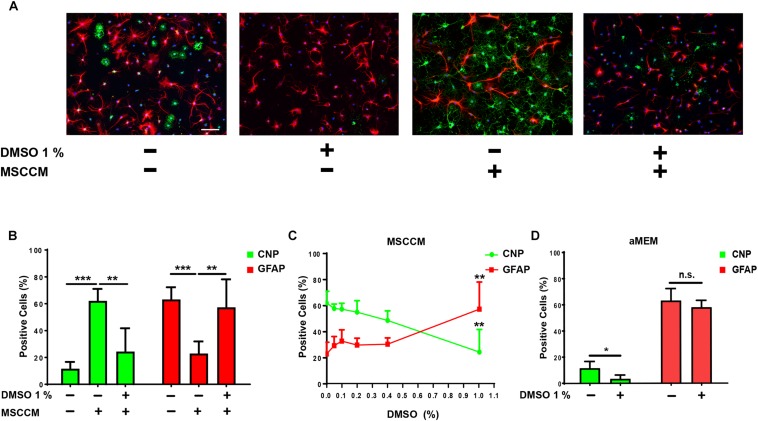
DMSO blocks the pro-oligodendrogenic effect of MSCCM and inhibits oligodendrocytic fate in NSPCs. **(A)** Representative immunodetection of CNP (green) and GFAP (red) expression in NSPCs differentiated for 6 days in aMEM and MSCCM, ±1% DMSO. Nuclei were counterstained with DAPI (blue). Note the decline of CNP^+^ cells in the presence of DMSO. Scale bar 100 μm. **(B)** Quantification of glial marker expression in NSPCs differentiated for 6 days in aMEM, MSCCM and MSCCM + 1% DMSO. **(C)** Analysis of glial marker expression in MSSCM with increasing concentrations of DMSO. **(D)** Quantification of glial marker expression in NSPCs differentiated for 6 days in aMEM and aMEM + 1% DMSO. Data represented as mean ± SD, ^∗^*p* < 0.05, ^∗∗^*p* < 0.01, ^∗∗∗^*p* < 0.001, n.s.: not significant.

To further evaluate the glial differentiation in these conditions, the expression of the astrocyte marker GFAP and the oligodendrocyte marker CNP were examined after 6 days of differentiation. In aMEM (*n* = 9), the majority of NSPCs differentiated into GFAP-expressing astrocytes, while the abundance of CNP^+^ oligodendrocytes remained similar to the level observed after 3 day of differentiation (CNP^+^ cells: 11.6 ± 5.0% and GFAP^+^ cells: 63.2 ± 9.0%) (Figures 2A,B). On the contrary, differentiation for 6 days in MSCCM (*n* = 9) significantly enhanced oligodendrogenesis, whereas astrogenesis was comparable to the level observed after 3 days (CNP^+^ cells: 62.1 ± 8.9% and GFAP^+^ cells: 22.9 ± 9.0%). The addition of 1% DMSO (*n* = 6) to differentiating cells nearly completely inhibited the MSCCM-induced oligodendrogenesis after 6 days (CNP^+^ cells: 24.4 ± 17.4%, *p* = 0.002; GFAP^+^ cells: 57.3 ± 20.8%, *p* = 0.008 as compared to MSCCM) (Figures 2A,B). We examined whether lower concentrations of DMSO also have an impact on the MSCCM pro-oligodendrogenic effect using DMSO concentrations of 0.05, 0.1, 0.2, 0.4, or 1% (*n* = 3; 3; 3; 3; 6, respectively). We observed that increasing concentrations of DMSO progressively inhibited the MSCCM-derived pro-oligodendrogenic activity, however, only a concentration of 1% DMSO significantly inhibited the oligodendroglial cell fate of differentiating NSPCs, in favor of astrocytes (Figure 2C).

Next, we extended our analyses to the spontaneous oligodendrocytic differentiation potential of NSPCs in aMEM to determine whether DMSO only neutralizes the activity of MSCCM or restricts the intrinsic capacity of NSPCs to undergo oligodendroglial differentiation. Following differentiation in aMEM with 1% DMSO (*n* = 3), the percentage of CNP-expressing NPSCs was significantly reduced as compared to cultures maintained in aMEM only (*n* = 9) (aMEM: 11.6 ± 5.0% compared to aMEM + 1% DMSO: 3.4 ± 2.8%; *p* = 0.011), whereas GFAP levels remained stable (aMEM: 69.4 ± 9.8% compared to aMEM + 1% DMSO: 58.1 ± 5.1%; *p* = 0.264) (Figures 2A,D). Furthermore, DMSO’s inhibitory effect on oligodendrogenesis was not limited to NSPCs from the hippocampal neurogenic niche, since NSPCs from the SVZ were affected in the same manner following addition of 1% DMSO during their differentiation (see Supplementary Figures S1A,B).

To exclude the possibility that the loss of oligodendrocytes results from an early selection through lineage-specific apoptosis, we quantified the frequency of active Caspase 3 in NG2+, Olig2+ or NG2/Olig2+ progenitor cells after 3 days of differentiation. Neither in aMEM, nor in MSCCM, the addition of 1% DMSO was leading to an increase in apoptosis in the subpopulations of oligodendrocyte progenitor cells (Supplementary Figure S3 and Supplementary Table S1). These results suggest that DMSO blocked the oligodendrogenic activity of MSCCM by suppressing the intrinsic capacity of NSPCs to undergo oligodendroglial differentiation in favor of an astrocytic fate.

### DMSO Alters the Balance of the Transcription Factors Olig2 and Id2

**FIGURE 3:**
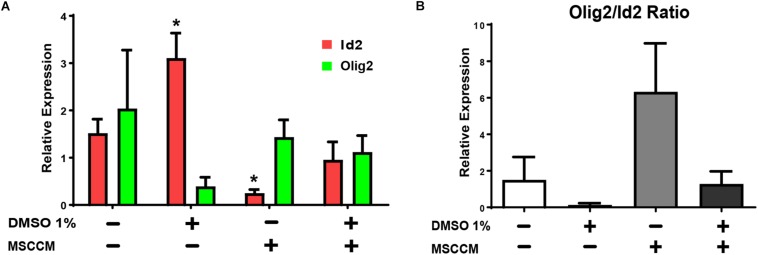
Expression of glial fate determinants after treatment with 1% DMSO. RT-PCRs of pro-oligodendrogenic Olig2 and pro-astrocytic Id2 gene expression were performed on NSPCs differentiating for 3 days. **(A)** Relative levels of mRNA expression for Id2 (red) and Olig2 (green) detected in NSPCs cultivated in aMEM or MSCCM, with or without 1% DMSO. **(B)** Ratio of Olig2/Id2. Data are shown as mean ± SD. Asterisks mark significant difference compared to aMEM, ^∗^*p* < 0.05.

The transcription factors Olig2 (oligodendroglial lineage) and Id2 (astroglial lineage) are key determinants of NSPC’s glial lineage commitment (Samanta and Kessler, 2004; Steffenhagen et al., 2012). Overexpression of Id2 can thereby lead to the inhibition of oligodendrogenesis and sequestration of Olig2 (Wang et al., 2001; Samanta and Kessler, 2004). To evaluate the gene expression of these glial fate determinants, we quantified the relative expression levels of Olig2 and Id2 in NSPCs after 3 days of differentiation in aMEM or MSSCM, with or without 1% DMSO (Figure 3A). In NSPCs exposed to MSCCM, Id2 expression levels were significantly reduced compared to aMEM (MSCCM: 0.3 ± 0.1, aMEM: 1.5 ± 0.3, *p* = 0.013, *n* = 3). Addition of 1% DMSO in MSCCM prevented this decrease (MSCCM + 1% DMSO: 1.0 ± 0.4; *p* = 0.117 compared to aMEM, *n* = 3). In aMEM on the other hand, DMSO induced an upregulation of Id2 mRNA (aMEM + 1% DMSO: 3.1 ± 0.5; *p* = 0.018 compared to aMEM, *n* = 3). The expression levels of Olig2 did not differ significantly throughout the conditions. Hence, these results suggest that DMSO mainly impacted differentiation through an increase of the expression of the astroglial fate determinant Id2, thereby inhibiting oligodendroglial fate decision in NSPCs.

In addition to their absolute levels of expression, the ratio between Olig2 and Id2 in the NSPCs is also crucial for fate decision. We have previously reported that pretreatment with MSCCM during proliferation enhanced the Olig2/Id2 ratio in NSPCs and this high ratio promotes an oligodendrocytic fate (Steffenhagen et al., 2012). Comparing the ratio calculated for various conditions used to differentiate the NSPCs, it is conspicuous that the addition of 1% DMSO, either in aMEM or in MSCCM, robustly decreased the Olig2/Id2 ratio, in correlation with the inhibition of the oligodendrogenic activity (Figure 3B).

### DMSO Reduces Phosphorylation of Erk1/2 in NSPCs During Differentiation

**FIGURE 4:**
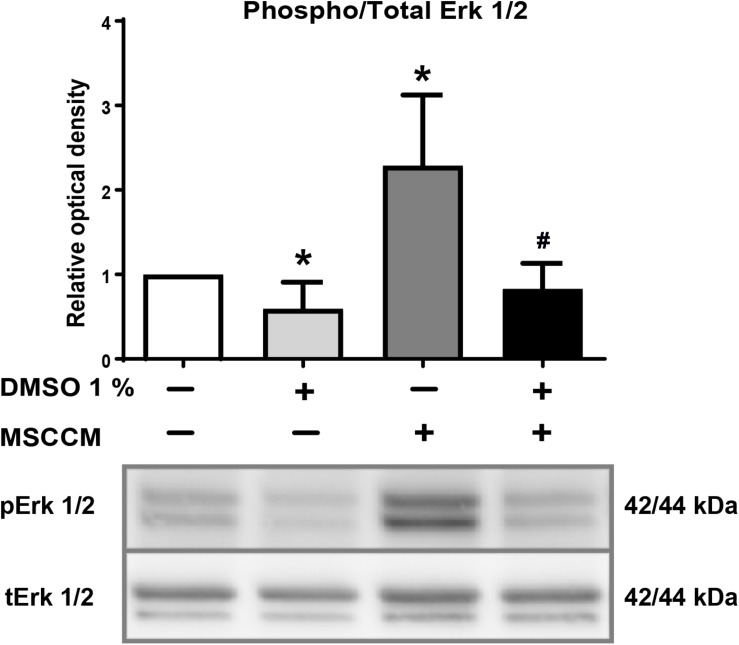
DMSO blocks phosphorylation of Erk1/2. Detection of phospho- and total Erk1/2 after exposure of NSPCs to DMSO. **(Upper panel)** The ratio of relative levels of pErk1/2 in relation to tErk1/2 quantified on western blots is shown for the various conditions. Addition of 1% DMSO significantly decreased phosphorylation of Erk1/2 in aMEM and MSCCM. Data represented as mean ± SD. Asterisks mark significant differences in relation to aMEM, pound sign marks significant differences in relation to MSCCM, ^∗^*p* < 0.05, #*p* < 0.05. **(Lower panel)** Representative western blots for tErk1/2 and pErk1/2 are shown.

Erk1/2 is activated by phosphorylation during oligodendrocyte differentiation (Fyffe-Maricich et al., 2011; Guardiola-Diaz et al., 2012). To evaluate whether the presence of DMSO modulates this crucial signaling pathway, we analyzed the level of Erk1/2 phosphorylation compared to total Erk1/2 (tErk1/2) in the various culture conditions. Erk1/2 phosphorylation, which is a strong oligodendrocytic fate inducer (Hu et al., 2004; Shi et al., 2014), was significantly elevated in NSPCs exposed to MSCCM (2.3 ± 0.8 fold change compared to aMEM, *p* = 0.026, *n* = 5) (Figure 4). In contrast, adding 1% DMSO to MSCCM reduced the levels of phosphorylated Erk1/2 (pErk1/2) back to the levels detected in NSPCs maintained in aMEM (MSCCM + 1% DMSO: 0.8 ± 0.3 fold change, *p* = 0.014 compared to MSCCM, *n* = 5). The lowest pErk1/2 levels were detected in aMEM with 1% DMSO (aMEM + 1% DMSO: 0.6 ± 0.3 fold change compared to aMEM, *p* = 0.044, *n* = 5). These results demonstrate the inhibition of a well-known oligodendrogenic pathway by the addition of DMSO (Figure 4).

## Discussion

For decades, DMSO has probably been the most utilized solvent for lipophilic agents in *in vitro* and *in vivo* studies. Due to its unique chemical properties, DMSO enables miscibility of hydrophobic substances and serves as a drug carrier enabling penetration into mammalian tissues. In addition, DMSO is widely used as cryoprotectant, e.g., for storage of stem cells. Unlike previous assumptions describing DMSO as inert in low concentrations, recent reports suggested an impact on the cell cycle, differentiation patterns, cell survival and epigenetic changes, even for concentration of DMSO below 1% (Santos et al., 2003; Iwatani et al., 2006). In the past years, additional studies have demonstrated that DMSO induces neurotoxic effects (Hanslick et al., 2009; Yuan et al., 2014), neurophysiological alterations in pyramidal neurons (Tamagnini et al., 2014) and activation of astrocytes (Zhang et al., 2017). Even though concerns over potential risks of using DMSO are growing, its impact on NSPCs residing in the CNS has not been carefully addressed yet.

In the present study, we investigated the impact of DMSO on the differentiation and glial fate decision of NSPCs. As previously reported (Rivera et al., 2006), under standard culture conditions (aMEM), the vast majority of NSPCs spontaneously differentiated into the astrocytic lineage, whereas addition of MSCCM strongly directed the fate choice toward oligodendrocytes. We could demonstrate that this pro-oligodendrogenic effect was inhibited to a large extent by the presence of DMSO during differentiation. This inhibitory activity of DMSO was not limited to NSPCs from the hippocampus, but also applied to NSPCs from the other adult neurogenic niche, i.e., the SVZ. We further observed a progressive inhibition of oligodendrogenesis with increasing concentrations of DMSO, however, significant inhibition in our *in vitro* conditions was observed at a concentration of 1% DMSO. As the detailed mode of action of DMSO on cell fate remains elusive, a significant inhibition of oligodendrogenesis at lower concentrations cannot be ruled out in the presence of additional factors or under pathological conditions.

We further investigated key determinants of oligodendrogenesis and found that the application of DMSO elevated the expression of Id2 in differentiating NSPCs cultivated either in aMEM, or in MSCCM. An overall decrease in the Olig2/Id2 ratio resulted in a shift of NSPCs fate decision away from oligodendrocytes and toward astrocytes. We also investigated the extracellular signal regulated kinases 1/2 (Erk1/2) pathway, which has been shown to regulate oligodendrogenic differentiation in NSPCs (Hu et al., 2004; Shi et al., 2014). We examined the phosphorylation status of Erk1/2 and detected a strong upregulation of the phosphorylated isoform following application of MSCCM. In contrast, the addition of DMSO in MSCCM blocked the phosphorylation of Erk1/2. Whether Erk1/2 dephosphorylation directly modulates Id2 expression under the influence of DMSO still needs to be evaluated.

Although remyelination is mainly achieved by OPC differentiation (Franklin and Goldman, 2015), NSPCs in the adult neurogenic niches have been shown to proliferate and migrate toward lesion sites and locally differentiate into oligodendrocytes as well (Menn et al., 2006; Etxeberria et al., 2010; Jablonska et al., 2010; Kazanis et al., 2017) As our results demonstrate strong anti-oligodendrogenic properties mediated by DMSO for cells of different neurogenic niches, even in the presence of MSCCM, the simultaneous application of MSCs and DMSO in transplantations might inhibit the MSC-mediated pro-oligodendrogenic activity. Furthermore, it would be of interest to evaluate, if OPC differentiation is similarly affected by DMSO, as documented in NSPCs, since upregulation of Id2 and dephosphorylation of Erk1/2 have been shown to block differentiation of OPCs as well (Wang et al., 2001; Fyffe-Maricich et al., 2011; Guardiola-Diaz et al., 2012).

In summary, our study demonstrates a robust anti-oligodendrogenic effect of DMSO on fate decision when present during NSPCs’ differentiation *in vitro*. Due to its broad use in cell culture, *in vivo* experiments and clinical trials, future studies evaluating the differentiation of NSPCs, oligodendrogenesis or remyelination should consider the impact of DMSO carefully and, whenever its removal is not possible, emphasize the use of appropriate controls.

## Data Availability Statement

All datasets generated for this study are included in the article/Supplementary Material.

## Ethics Statement

Ethical review and approval was not required for the animal study because the legislation does not require ethics approval for the killing of animals with the aim of collecting tissues.

## Author Contributions

AO’S, SL, PR, and LB conducted and analyzed the experiments. AO’S, PR, and LB performed the statistical analysis. AO’S, FR, and SC-D wrote the manuscript. All authors contributed to the conception, design, and finalizing of the study, and approved the submitted version.

## Conflict of Interest

The authors declare that the research was conducted in the absence of any commercial or financial relationships that could be construed as a potential conflict of interest.

## Supplementary Material

FIGURE S1 SVZ and HC NSPCs react equally to DMSO exposure. Quantification of glial marker expression in NSPCs differentiated for 6 days in aMEM (HC *n* = 9, SVZ *n* = 3), aMEM + 1% DMSO (HC *n* = 3; SVZ *n* = 3), MSCCM (HC *n* = 9; SVZ *n* = 3) and MSCCM + 1% DMSO (HC *n* = 6; SVZ *n* = 3). Comparison of HC and SVZ NSPCs marker expression: **(A)** CNP and **(B)** GFAP. No statistical difference was detected between the two groups. Data represented as mean ± SD, n.s.: not significant.

FIGURE S2 DMSO and MSCCM decrease DCX expression. RT-PCRs of DCX gene expression were performed on NSPCs differentiating for 3 days. Relative mRNA expression level of DCX detected in NSPCs cultivated in aMEM or MSCCM, with or without 1% DMSO respectively. Data are shown as mean ± SD. Asterisks mark significant difference compared to aMEM, ^∗^*p* < 0.05, n.s.: not significant.

FIGURE S3 Detection of Caspase 3 after 3 days of differentiation. Representative immunodetection of NG2 (green), Olig2 (white) and Caspase3 (red) in NSCs after 3 days of differentiation in **(A)** aMEM, **(B)** aMEM + 1% DMSO, **(C)** MSCCM, and **(D)** MSCCM + 1% DMSO. Nuclear counterstained with DAPI (blue). Scale bar in **D**: 100 μm. Example of cells positive for Caspase3 are identified with arrows.

TABLE S1 DMSO does not induce specific cell death in cells of oligodendrocyte lineage. Percentages of NG2/Casp3^+^, Olig2/Casp3^+^ and NG2/Olig2/Casp3^+^ cells in relation to their respective population (NG2^+^, Olig2^+^, NG2/Olig2^+^) after 3 days of differentiation. Percentages of CNP/Casp3+ and GFAP/Casp3+ cells in relation to their respective population (CNP+ and GFAP+). *P*-values comparing fractions of Casp3^+^ cells of specific NG2^+^, Olig2^+^ and NG2/Olig2^+^ cell populations in aMEM or MSCCM with and without 1% DMSO. Data represented as mean ± SD. *n* = 3. ND: not determined. DMSO did not significantly affected Casp3 frequency in the subpopulations.
