# Supplementary figures and images for: Dimethylsulfoxide Inhibits Oligodendrocyte Fate Choice of Adult Neural Stem and Progenitor Cells
Source: Front Neurosci. 2019 Nov 26;13:1242. doi: 10.3389/fnins.2019.01242 (PMC6901908; doi:10.3389/fnins.2019.01242)

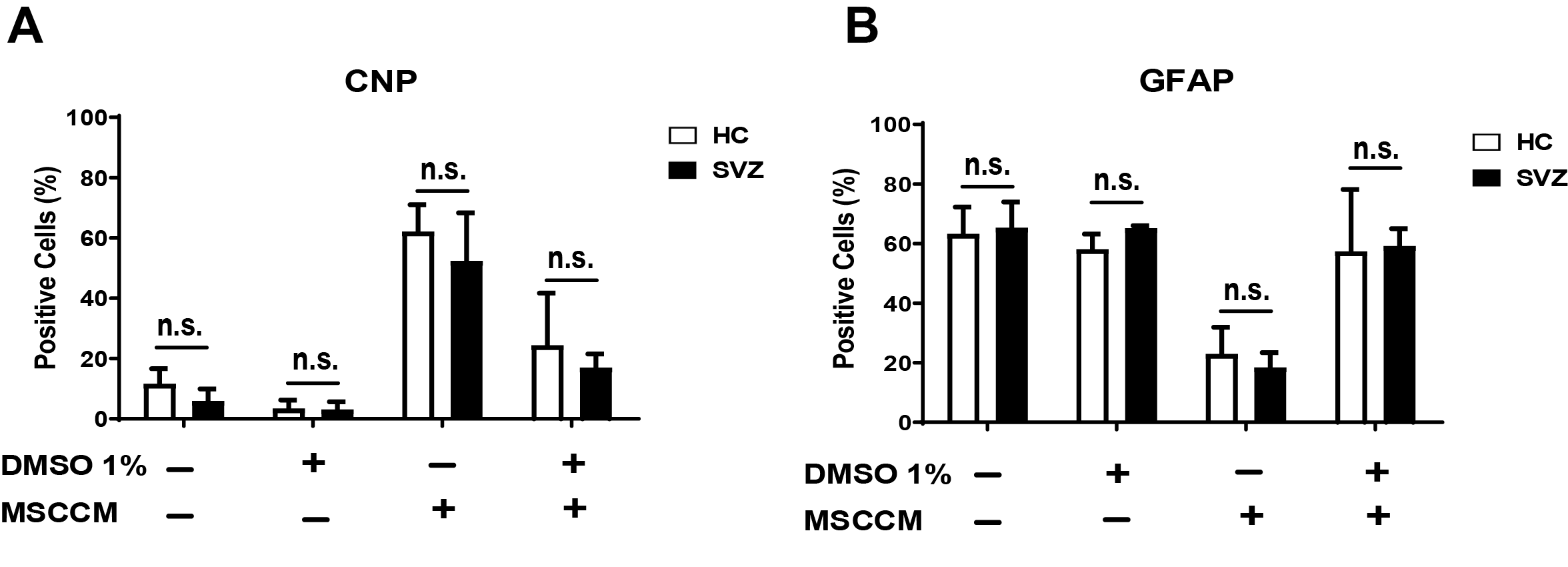

Supplement: FIGURE S1 — SVZ and HC NSPCs react equally to DMSO exposure. Quantification of glial marker expression in NSPCs differentiated for 6 days in aMEM (HC n = 9, SVZ n = 3), aMEM + 1% DMSO (HC n = 3; SVZ n = 3), MSCCM (HC n = 9; SVZ n = 3) and MSCCM + 1% DMSO (HC n = 6; SVZ n = 3). Comparison of HC and SVZ NSPCs marker expression: (A) CNP and (B) GFAP. No statistical difference was detected between the two groups. Data represented as mean ± SD, n.s.: not significant. [file Image_1.TIF]

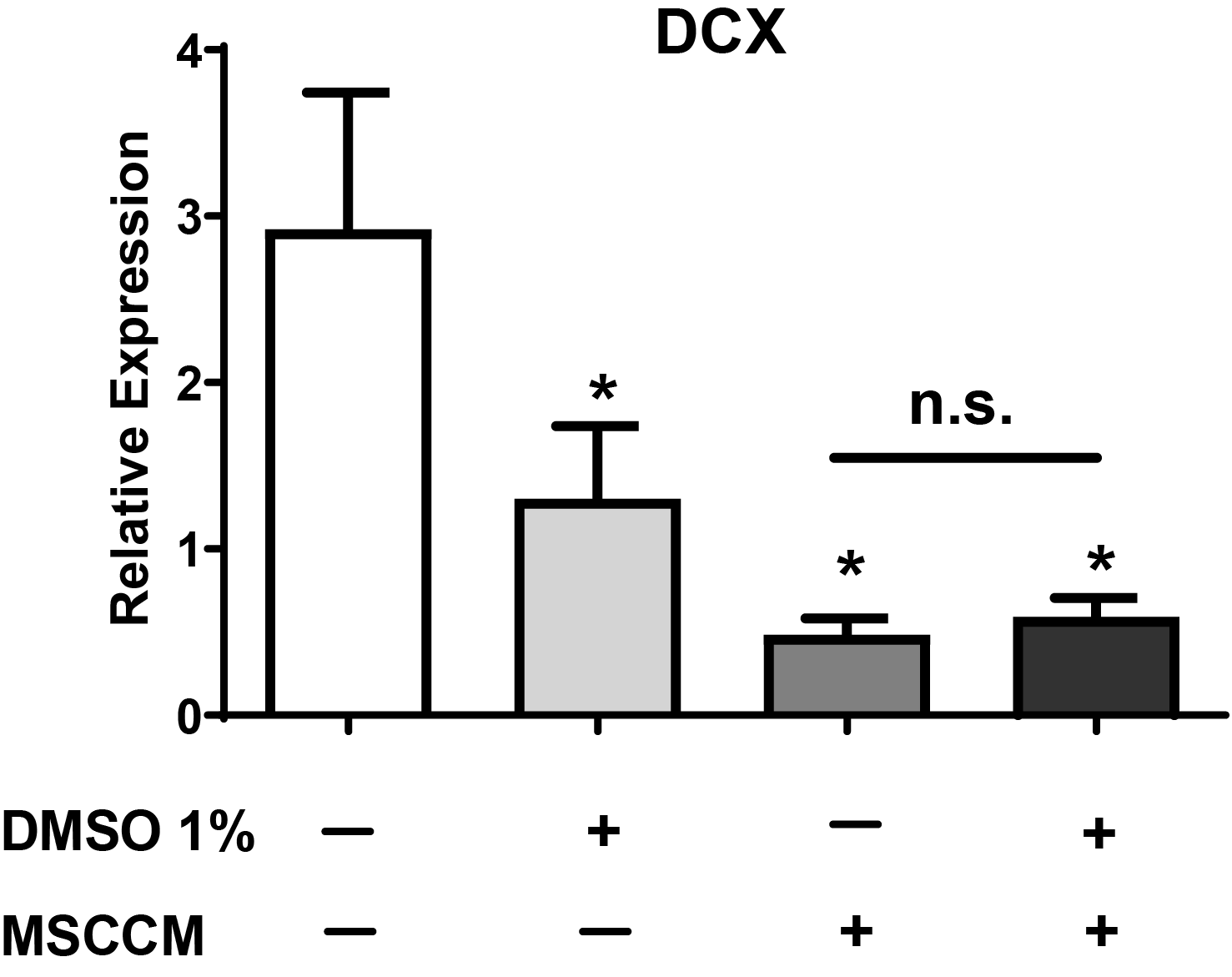

Supplement: FIGURE S2 — DMSO and MSCCM decrease DCX expression. RT-PCRs of DCX gene expression were performed on NSPCs differentiating for 3 days. Relative mRNA expression level of DCX detected in NSPCs cultivated in aMEM or MSCCM, with or without 1% DMSO respectively. Data are shown as mean ± SD. Asterisks mark significant difference compared to aMEM, ∗p < 0.05, n.s.: not significant. [file Image_2.TIF]

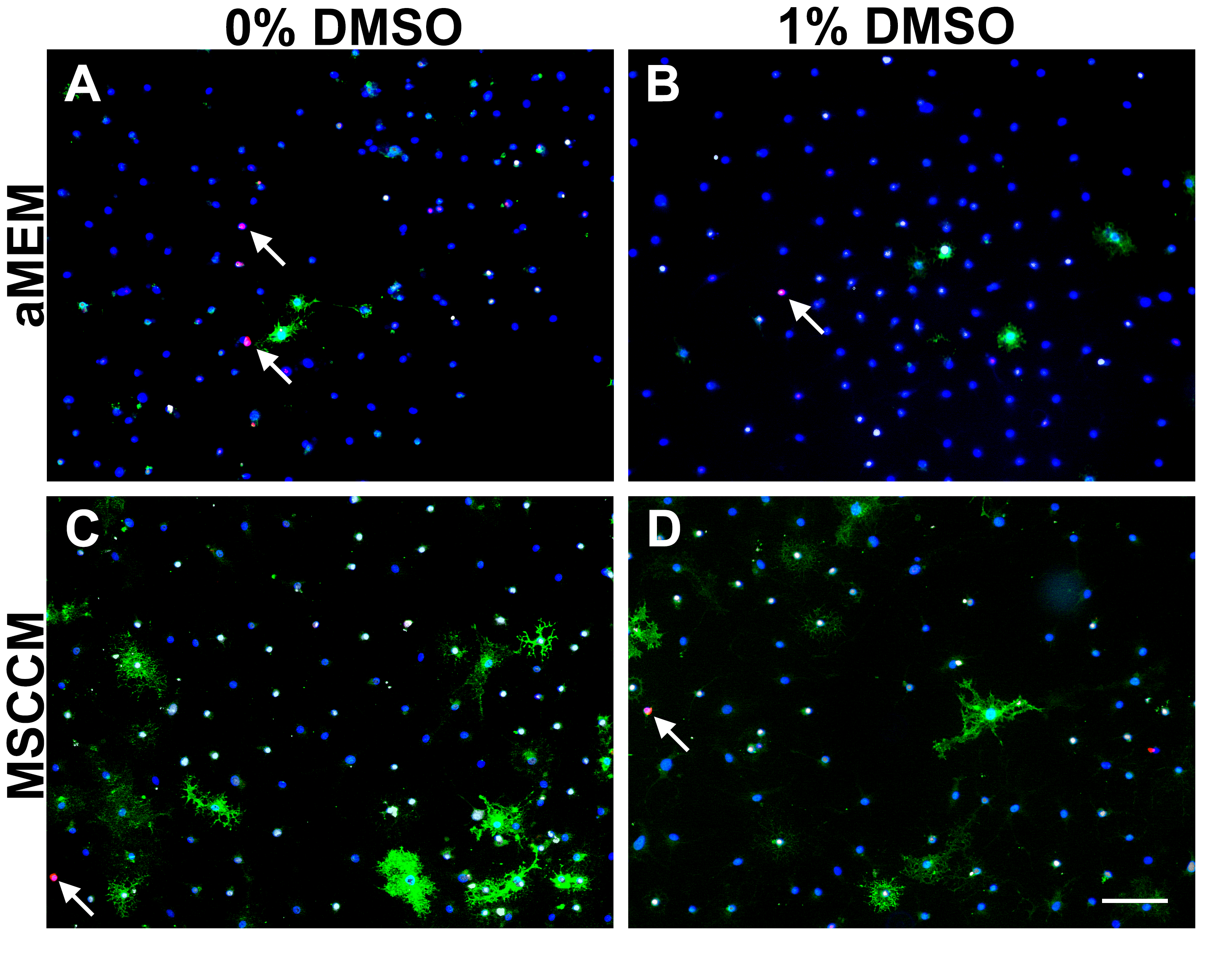

Supplement: FIGURE S3 — Detection of Caspase 3 after 3 days of differentiation. Representative immunodetection of NG2 (green), Olig2 (white) and Caspase3 (red) in NSCs after 3 days of differentiation in (A) aMEM, (B) aMEM + 1% DMSO, (C) MSCCM, and (D) MSCCM + 1% DMSO. Nuclear counterstained with DAPI (blue). Scale bar in D: 100 μm. Example of cells positive for Caspase3 are identified with arrows. [file Image_3.TIF]
