# Supplementary material for: Dimethylsulfoxide Inhibits Oligodendrocyte Fate Choice of Adult Neural Stem and Progenitor Cells
Source: Front Neurosci. 2019 Nov 26;13:1242. doi: 10.3389/fnins.2019.01242 (PMC6901908; doi:10.3389/fnins.2019.01242)
Supplement: TABLE S1 — DMSO does not induce specific cell death in cells of oligodendrocyte lineage. Percentages of NG2/Casp3+, Olig2/Casp3+ and NG2/Olig2/Casp3+ cells in relation to their respective population (NG2+, Olig2+, NG2/Olig2+) after 3 days of differentiation. Percentages of CNP/Casp3+ and GFAP/Casp3+ cells in relation to their respective population (CNP+ and GFAP+). P-values comparing fractions of Casp3+ cells of specific NG2+, Olig2+ and NG2/Olig2+ cell populations in aMEM or MSCCM with and without 1% DMSO. Data represented as mean ± SD. n = 3. ND: not determined. DMSO did not significantly affected Casp3 frequency in the subpopulations. [file Table_1.docx]

**Table 1. *Caspase 3 detection in the various cell populations***

|  | | **aMEM** | | | **MSCCM** | | |
| --- | --- | --- | --- | --- | --- | --- | --- |
|  | **DMSO 1%** | **-** | **+** | **p-value** | **-** | **+** | **p-value** |
| **NG2/Casp3+** |  | 10.86±2.06% | 11.04±0.51% | 0.895 | 6.28±3.22% | 9.63±3.74% | 0.307 |
| **Olig2/Casp3+** |  | 15.92±6.85% | 19.75±6.25% | 0.514 | 5.21±3.23% | 8.95±6.49% | 0.439 |
| **NG2/Olig2/Casp3+** |  | 21.23±5.29% | 18.26±1.53% | 0.436 | 8.08±5.59% | 14.28±6.08% | 0.273 |
| **CNP/Casp3+** |  | 8.92±1.44% | ND | ND | 4.21±3.74% | 5.63±1.90% | 0.590 |
| **GFAP/Casp3+** |  | 22.54±1.53% | ND | ND | 9.49±2.77% | 7.34±3.76% | 0.471 |

**Table 1:** DMSO does not induce lineage specific cell death in oligodendrocyte precursor cells.

Percentages of NG2/Casp3^+^, Olig2/Casp3^+^ and NG2/Olig2/Casp3^+^ cells in relation to their respective population (NG2^+^, Olig2^+^, NG2/Olig2^+^) after 3 days of differentiation. Percentages of CNP/Casp3^+^ and GFAP/Casp3^+^ cells in relation to their respective population (CNP^+^ and GFAP^+^). P-values comparing fractions of Casp3^+^ cells of specific populations in aMEM or MSCCM with and without 1% DMSO. Data represented as mean ± SD, n=3. ND: not determined.
